# Robotic oncologic colorectal surgery with a new robotic platform (CMR Versius): hope or hype? A preliminary experience from a full-robotic case-series

**DOI:** 10.1007/s10151-022-02626-9

**Published:** 2022-05-30

**Authors:** Cristiano Huscher, Francesco Marchegiani, Francesco Cobellis, Patricia Tejedor, Carlos Pastor, Gianni Lazzarin, James Wheeler, Salomone Di Saverio

**Affiliations:** 1 Casa di Cura Cobellis, Vallo della Lucania, Salerno, Italy; 2 Department of Surgical Oncology, Robotics and New Technologies, Policlinico Abano, Padua, Italy; 3 Department of Surgical, Oncological and Gastroenterological Sciences, University of Padua, Padua, Italy; 4 University of Padua, Padua, Italy; 5 Department of Colorectal Surgery, University Hospital Gregorio Marañon, Madrid, Spain; 6 Department of Colorectal Surgery, University Clinic of Navarre, Madrid-Pamplona, Spain; 7 Cambridge University Hospitals NHS Foundation Trust, Cambridge, UK; 8 General Surgery Department “Paride Stefanini”, La Sapienza University of Rome, Rome, Italy; 9 Department of General Surgery, Madonna del Soccorso Hospital, Asur Marche Area Vasta 5, San Benedetto del Tronto, Ascoli Piceno, Italy; 10 Brighton and Sussex University Medical School , Brighton, UK

**Keywords:** Colorectal cancer, Robotic surgery, Minimally invasive surgery, Robotics

## Abstract

**Background:**

The present case-series describes the first full-robotic colorectal resections performed with the new CMR Versius platform (Cambridge Medical Robotics Surgical, 1 Evolution Business Park, Cambridge, United Kingdom) by an experienced robotic surgeon.

**Methods:**

In a period between July 2020 and December 2020, patients aged 18 years or older, who were diagnosed with colorectal cancer and were fit for minimally invasive surgery, underwent robotic colorectal resection with CMR Versius robotic platform at “Casa di Cura Cobellis” in Vallo della Lucania,Salerno, Italy. Three right colectomies, 2 sigmoid colectomies and 1 anterior rectal resection were performed. All the procedures were planned as fully robotic. Surgical data were retrospectively reviewed from a prospectively collected database.

**Results:**

Four patients were male and 2 patients were female with a median (range) age of 66 (47–72) years. One covering ileostomy was created. Full robotic splenic flexure mobilization was performed. No additional laparoscopic gestures or procedures were performed in this series except for clipping and stapling which were performed by the assistant surgeon due to the absence of robotic dedicated instruments. Two ileocolic anastomoses, planned as robotic-sewn, were performed extracorporeally. One Clavien–Dindo II complication occurred due to a postoperative blood transfusion. Median total operative time was 160 (145–294) min for right colectomies, 246 (191–300) min for sigmoid colectomies and 250 min for the anterior rectal resection.

**Conclusions:**

The present series confirms the feasibility of full-robotic colorectal resections while highlighting the strengths and the limitations of the CMR Versius platform in colorectal surgery. New devices will need more clinical development to be comparable to the current standard.

**Supplementary Information:**

The online version contains supplementary material available at 10.1007/s10151-022-02626-9.

## Introduction

Robotic surgery has been expanding all over the world over the last two decades. As a consequence, colorectal surgery has experienced important technical advances even if currently no strong advantages in terms of short-term nor long-term outcomes are evident [1]. Recently, Intuitive Surgical DaVinci^®^ systems have been challenged by the recent arrival of new robotic platforms on the market.

CMR Versius robot (Cambridge Medical Robotics Surgical, 1 Evolution Business Park, Cambridge, United Kingdom) is a new teleoperated surgical robotic system designed in Cambridge (UK). This new device obtained European Conformity (CE) mark approval in March 2019. In the autumn of 2019, two different systems began to be used in a clinical setting in India and the United Kingdom and in 2020 the first Australian installation was reported. In the last 2 years, different countries in Europe adopted CMR Versius in general surgery, urology and gynaecology. In Italy, the first CMR Versius robotic platform was installed in Vallo della Lucania (Salerno), in 2020.

Despite the broad adoption of this new platform, few reports were published concerning its use in colorectal surgery. Dixon et al. [2] and Collins et al. [3] recently published the first two case-series on this topic.

The authors reported hybrid procedures (robotic and laparoscopic) demonstrating the feasibility of colorectal surgery and the safety of this new robotic device. They showed that this platform has been positively evaluated in those centres where no prior robotic surgery had previously been performed.

The aim of the present study is to report our first case-series outcomes of colorectal cancer resections performed fully robotically using the CMR Versius Robotic platform. All the procedures were performed by a surgeon who has previous extensive experience in robotic surgery (C. H.).

## Materials and methods

### Reporting

The present article follows the Preferred Reporting Of CasE Series in Surgery (PROCESS) checklist belonging to the EQUATOR Network site [4, 5].

### Ethical issues and informed consent

Informed consent was obtained by patients who underwent robotic surgery with Versius robot at “Casa di Cura Cobellis” in Vallo della Lucania (Salerno, Italy) Ethical standards were respected in compliance with the 2013 updated Helsinki Declaration concerning ethical principles for medical research involving human subjects [6]. Anonymized data were retrospectively reviewed from a prospectively collected database provided by the CMR company and filed by the responsible surgeon with the supervision of the company engineer.

### Robotic platform

**Fig. 1:**
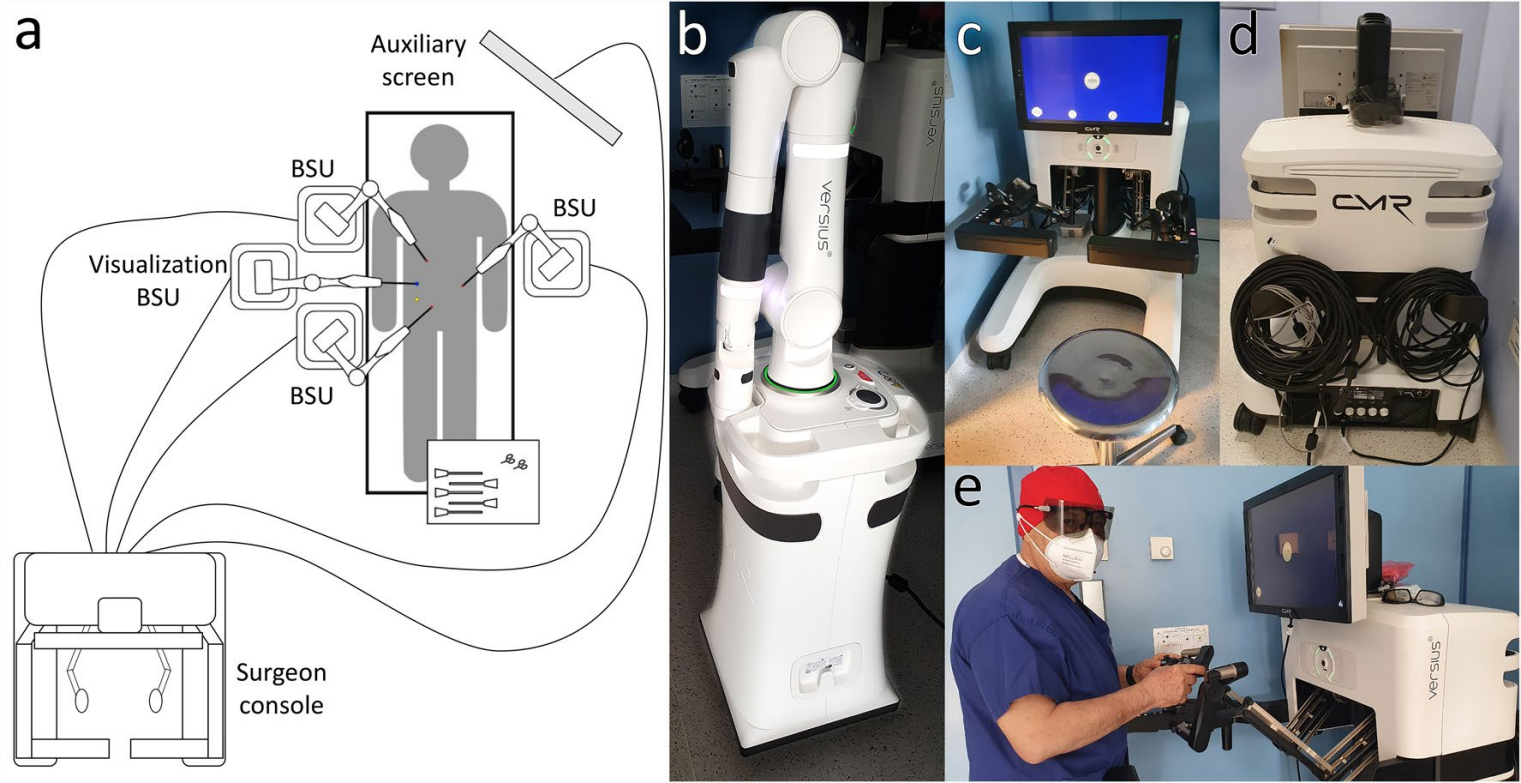
Versius robotic platform. **a** Diagram of the system. The master console is wire connected to the independent bedside units (BSUs). One BSU is dedicated to the camera (visualization BSU). The auxiliary screen is connected to the console. **b** View of a single BSU. **c** Front view of the surgeon console. **d** Rear view of the surgeon console with the connection wires. **e** Surgeon at the console in standing position

The CMR Versius robotic platform is composed of a master console and 3 to 4 independent bedside units (BSUs) which are wire connected to the console. (Fig. 1) A fifth BSU is available for future technological developments but is not currently adoptable during surgery.

The master console guarantees a three-dimensional view with the use of passive polarized glasses. The device is completely controlled by hands so it can be used in both seated and standing positions. The controller is composed of a handle containing: a lever for instrument jaw opening, a clutch button, an energy activation button, a joystick for endoscope control and an energy led indicator. One joystick controls rotation and distance of the camera while the other one controls the movements of the camera arm [7]. The BSUs have a limited weight (100 kg) and are not provided with engines so they can be positioned manually. Before the platform docking, every single BSU must be covered by a sterile drape. Once the position around the patient is reached, a button activates the brake which stabilizes the BSU on the floor. Then, 12-mm endoscope and 5-mm instruments can be properly mounted. Once in place, the instruments are rotated in the trocar in a cone-like fashion completing a process called “port training”. No specific trocars are needed but balloon-cuffed ones are recommended to avoid their displacement. No specific insufflators or energy devices are needed.

### Surgeon

This series comes from a single surgeon (C.H.) who is a pioneer in the field of minimally invasive surgery and who has been performing robotic surgery for 20 years. This experience started in 2001 with the Computer Motion Zeus robotic surgical system which was then abandoned in favour of the Intuitive Surgical DaVinci^®^. The operating surgeon has completed more than 1500 robotic procedures in different settings (public hospitals and private clinics).

The surgeon and the assistants were properly trained by the company obtaining a proficiency certificate based on simulation, dry lab and cadaver lab [8]. All the procedures were performed under the supervision of the company team composed of engineers as part of an implementation program.

### Patients

In the period between July 2020 and December 2020, patients aged 18 years or older, who were diagnosed with resectable colorectal cancer and were fit for minimally invasive surgery, underwent robotic colorectal resection with the CMR Versius robotic platform.

### Procedures

A total of 6 procedures, 3 right colectomies, 2 sigmoid colectomies and 1 anterior rectal resection were performed. All the procedures were planned as fully robotic to maximize the possible efficacy of the platform. The performed procedures were not-consecutive cases.

#### Port placement

**Fig. 2:**
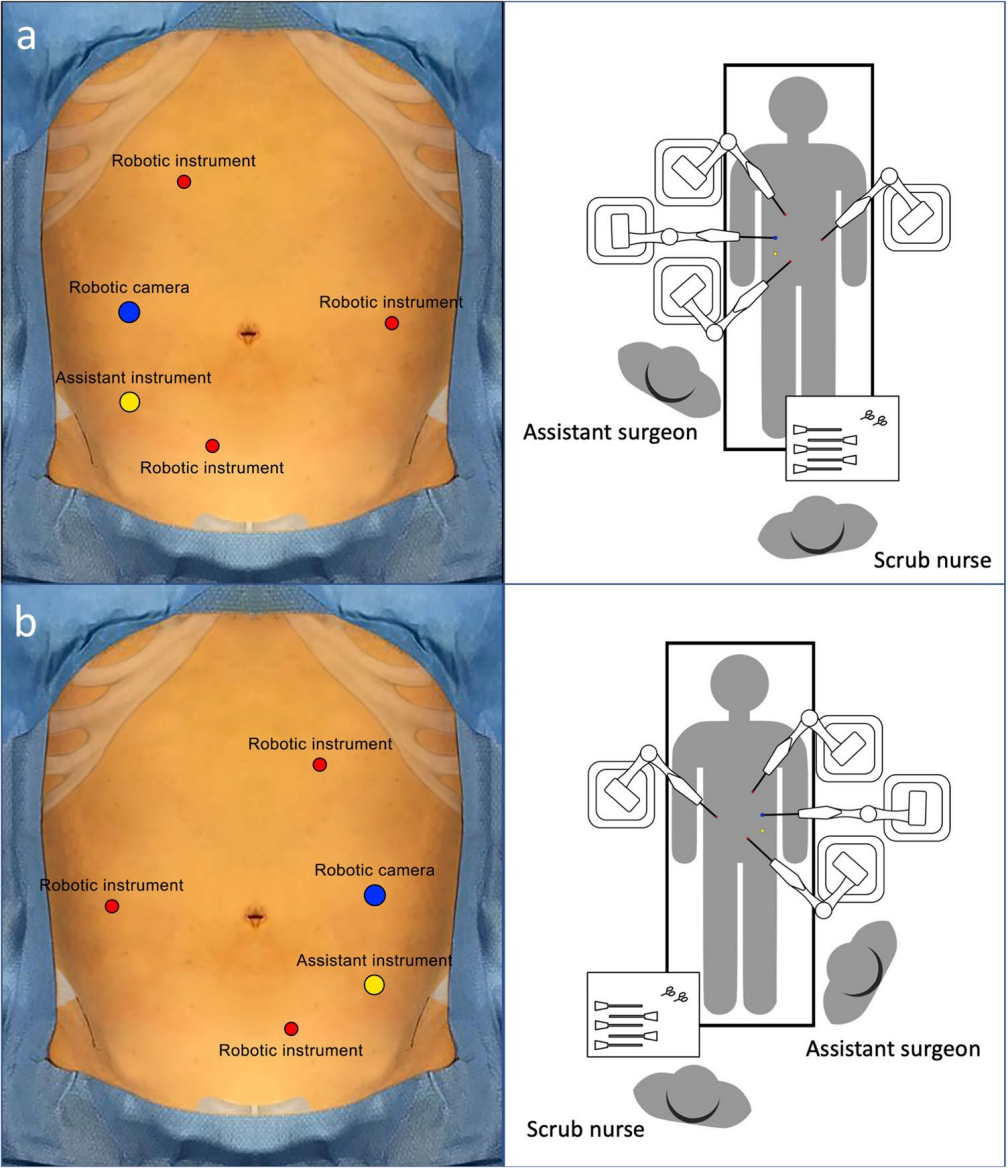
Trocar and bedside unit positioning. **a** Setting for sigmoid colectomy and anterior rectal resection. **b** Setting for right colectomy

Port placement and BSU placement (Fig. 2) were based on laparoscopic experience and technical advice from the company. Minor variations of the BSU positions were performed during the procedure due to the conflict of the robotic arms, known as “clashing”.

#### Instruments and disposables

Interventions were performed by adopting all the available robotic instruments (monopolar scissor, Maryland bipolar forceps, monopolar hook, fenestrated grasper and needle holder). The assistant surgeon adopted advanced bipolar devices, metallic clips, Hem-o-lok^®^ clips (Weck, Teleflex Inc. 550 E. Swedesford Road Suite 400. Wayne, PA, USA) and Echelon Flex™ electrical powered linear staplers (Ethicon, Johnson and Johnson, 1 Johnson And Johnson Plaza, New Brunswick, NJ, USA).

All the anastomoses performed after right colectomies were performed with Stratafix^®^ barbed suture (Johnson and Johnson, 1 Johnson And Johnson Plaza, New Brunswick, NJ, USA).

All the anastomoses performed after sigmoid colectomies or rectal resection where mechanical Knight-Griffen anastomoses performed with a circular stapler ILS (Ethicon, Johnson and Johnson, 1 Johnson And Johnson Plaza, New Brunswick, NJ, USA).

All the mini-laparotomies were protected by an Alexis™ wound protector (Applied Medical Technology Inc. 8006 Katherine Blvd, Brecksville, OH, USA). No surgical drains were placed at the end of any of the procedures.

#### Right colectomy

After a proper ileocecal retraction with the fourth robotic arm, the ileocecal vessels were exposed and ligated at their origin from the superior mesenteric vessels. The dissection proceeded in a medial-to-lateral direction along the avascular plane between the Toldt and Gerota fascia until the complete mobilization of the right colon. Then, the last ileal loop was divided with a linear stapler. In case of the ascending colon location, the right branches of the middle colic vessels were exposed and divided. The transverse colon was divided using linear staplers. An ileocolic intra-corporeal isoperistaltic side-to-side robotic-sewn anastomosis was fashioned when possible, as previously described. Otherwise, the same anastomosis was performed extracorporeally through a mini-laparotomy. The specimen was retrieved through a mini-laparotomy covered by a wound protector (Video 1).

#### Sigmoid colectomy and anterior rectal resection

After sigmoid retraction with the fourth robotic arm, the inferior mesenteric artery was exposed and isolated. The vessel was divided at its origin (high-tie ligation). The dissection proceeded in a medial-to-lateral direction along the avascular plane between the Toldt and Gerota fascia until the complete mobilization of the left colon. Splenic flexure mobilization was routinely performed. The inferior mesenteric vein was preserved when possible otherwise it was ligated at its origin. Rectal transection was performed at the level of the peritoneal reflection in case of sigmoid cancer while a dissection in the mesorectal plane was performed in case of upper rectal cancer with a subsequent lower rectal transection. The colonic stump was extracted through a mini-laparotomy covered by a wound protector and the colon was sectioned at the established point. Then, the anvil was inserted in the colonic stump and a Knight–Griffen colorectal anastomosis was performed. In case of rectal resection, a loop ileostomy was performed to protect the anastomosis (Video 2).

### Outcomes

Demographic, intraoperative and postoperative data were collected. Data fields included sex, age, body mass index (BMI), comorbidities, American Society of Anesthesiologists (ASA) score, previous abdominal surgery, previous chemotherapy or radiotherapy, previous stenting, technique of pneumoperitoneum induction, trocar placement scheme, methods of vessels ligation, accessory energy device adoption, number of staplers adopted, extraction site, type of anastomosis, flexure mobilization, stoma creation, conversion to laparoscopy or laparotomy, reason for conversion, blood loss, intraoperative and postoperative blood transfusion, procedural time from skin incision to skin closure, first flatus date, length of hospital stay, histopathology, TNM, number of positive lymph nodes, number of retrieved lymph nodes, complications within 30 days according to the Clavien–Dindo classification [9], unplanned readmissions within 30 days. Results are presented as median (range).

### Statistical analysis

Descriptive statistics were reported as median with range for continuous variables and absolute numbers (percentages) for categorical variables. All the extracted data were managed using Microsoft Excel.

## Results

The patients’ baseline characteristics and tumour locations are presented in Table 1. Five patients (83.3%) had had previous abdominal surgeries, and median BMI was 24.9 kg/m^2^. We reported the surgical procedures, intraoperative details and short-term morbidity in Table 2; 3 right colectomies, 2 sigmoid colectomies and 1 anterior resection were performed fully robotically. Two out of three ileocolic anastomoses which were planned as intra-corporeal robotic-sewn, were performed extracorporeally. One case was related to the poor dexterity of the platform while the other occurred due to an emergency that required the first surgeon to leave the surgical theatre leaving the assistant constructing the anastomosis. No part of the surgery was performed laparoscopically instead of robotically except for clipping and stapling, which was done by the assistant surgeon due to the current lack of dedicated instruments. For the latter reason, the assistant completed the vessel dissection in 4 cases with the aid of an advanced energy device. The left-colic and rectal surgeries required a full mobilization of the splenic flexure that was also performed robotically. The patient who underwent rectal cancer surgery required a protective ileostomy.

**Table 1:** Patient characteristics

	No. of patients (*n* = 6)
Age (years)^a^	66 (47–72)
Sex	
Female	2
Male	4
Body mass index (kg/m^2^)^a^	24.9 (23.2–33.9)
CCI^a^	5 (2–8)
ASA score	
II	4
III	2
Previous abdominal surgery	5
Tumour location	
Coecum	2
Ascending colon	1
Sigmoid colon	2
Upper rectum	1
Previous chemotherapy	0
Previous stenting	0

*CCI* Charlson comorbidity index, *ASA* American Society of Anesthesiologists

^a^Values are expressed as median (range)

**Table 2:** Surgical procedures

Patient number	Intervention	Pneumoperitoneum induction technique and location	Vessels ligation	Vessels ligation device	Advanced bipolar adoption	Anastomosis	Conversion to laparotomy	Extraction site	Blood loss (ml)^a^
1	Right colectomy	Veress needle left hypocondrium	ICA; ICV + rbMCA; rbMCV	Vascular stapler; Hem-o-lok	No	Extracorporeal Isoperistaltic Hand-sewn	No	Transverse incision – right hypocondrium	101–500
2	Right colectomy	Hasson open umbilical	ICA; ICV + rbMCA; rbMCV	Vascular stapler; Hem-o-lok	Yes	Intracorporeal Isoperistaltic Robotic-sewn	No	Transverse incision – right hypocondrium	0–100
3	Right colectomy	Veress needle left hypocondrium	ICA; ICV	Hem-o-lok	Yes	Extracorporeal Isoperistaltic Hand-sewn	No	Transverse incision – right hypocondrium	0–100
4	Sigmoidectomy	Veress needle left hypocondrium	IMA; IMV	Hem-o-lok	No	Knight-Griffen	No	Suvrapubic Pfannenstiel incision	0–100
5	Sigmoidectomy	Hasson open umbilical	IMA	Vascular stapler	Yes	Knight-Griffen	No	Suvrapubic Pfannenstiel incision	0–100
6	Anterior rectal resection + ileostomy	Veress needle left hypocondrium	IMA	Hem-o-lok	Yes	Knight-Griffen	No	Transrectal incision – right flank	0–100

*ICA* ileocolic artery, *ICV* ileocolic vein, *rbMCA* right branches of the middle colic artery, *rbMCV* right branches of the middle colic vein, *IMA* inferior mesenteric artery, *IMV* inferior mesenteric vein

^a^Recorded and reported as range

The median total operative time was 160 (145–294) min for right colectomies, 246 (191–300) min for sigmoid colectomies and 250 min for the anterior rectal resection. No intraoperative blood loss was recorded except for the first operated patient who had between 100 and 500 ml blood loss during a right colectomy. Short-term outcomes and histopathological results are summarized in Table 3. The median time to first flatus was 2.5 (2–3) days. No complications occurred in the 30 days after intervention except for a minor complication (Clavien–Dindo II) due to the previously mentioned blood transfusion. No re-interventions were required and no unplanned readmissions were recorded. Median hospital stay was 6.5 (5–7) days. The histopathological report showed a complete R0 resection in every patient with a median lymph node yield of 13 (12–15) lymph nodes. The mesorectal excision was considered complete by the pathologist (according to the College of American Pathologists). Two patients received adjuvant chemotherapy.

**Table 3:** Short-term outcomes and histopathology

Patient number	Blood transfusions (POD)	First flatus	LOS	Clavien–Dindo classification	Histopathology	Lymph nodes (positive/total)
1	No	POD 3	6	–	Adenocarcinoma G2—pT2N0M0	0/15
2	Yes—POD 1	POD 2	7	2	Adenocarcinoma G2—pT3N0M0	0/15
3	No	POD 3	7	–	Adenocarcinoma G2—pT3N0M0	0/13
4	No	POD 2	5	–	Adenocarcinoma G2—pT3N0M0	0/12
5	No	POD 2	6	–	Adenocarcinoma G2—pT4aN1cM0	0/12
6	No	POD 3	7	–	Adenocarcinoma G2—pT3N1aM1a	1/13

*POD* postoperative day, *LOS* length of stay, *G* grading

## Discussion

The present study shows the feasibility of the CMR Versius robotic platform in performing fully robotic elective colorectal cancer surgeries.. To our knowledge, this is the first case-series performed with the full adoption of the CMR Versius platform without modifying the surgical technique when compared to conventional robotic surgery.Our short-term outcomes and the oncological results were comparable to other previously published series using the same robotic system [2, 3].

New surgical platforms are entering the medical market and the coming years will be characterized by novelties in this sector. Whether these innovations are useful for surgical oncologists is still matter of debate. Despite some previous attempts to offer a valid alternative for robotic surgery [10, 11], CMR Versius is the first European commercial competitor of the DaVinci^®^ robotic platform.

Previously reported series on colorectal surgery performed with the CMR robotic platform, were conducted in a robot-naïve environment [2, 3]. All the procedures had a predefined limited robotic console time and they implied the adoption of laparoscopy to complete some tasks as flexure mobilization, pelvic dissection or anastomosis completion.

In robotic surgery, technology is crucial and the gain in procedural costs can be justified when the platform is fully exploited for all the surgical steps from dissection until reconstruction [12–14].

When analysing the history of the robotic surgery performed with the DaVinci^®^ platform, two factors are observed to be responsible for the operative time saving: the learning curve and the robot evolution, which is mainly related to the different docking time of the last generation, namely DaVinci^®^ Xi™.

However, as recently demonstrated by a meta-analysis comparing robotic and laparoscopic right colectomies, both with intra-corporeal and extra-corporeal anastomosis, the Achilles’ heel of robotic surgery remains the longer operative time [15].

In the present series, the median overall operative time for right colectomies was 160 min. It exceeded the 130 min reported for robotic conventional right colectomies in a previously published series [16].

Nevertheless, the first adoption of a new robotic platform requires optimization and completion of the learning curve even for an experienced surgeon [17] and these results cannot be considered conclusive. Unfortunately, the main limitation of this study is the absence in the registry of specific time measurement of all the surgical steps (trocar positioning and setup time, docking time, operative time, clashing time, final closure time). However, it is evident when performing a retrospective video review, that the main aspects to improve are the docking which is time consuming when compared to DaVinci^®^ Xi™ platform [16] and the clashing.

The independence of the robotic arms is the most versatile characteristic of the Versius robot but at the same time the clashing becomes the most limiting factor, as previously reported [3]. No clinical suggestions were given by the company to reduce this inconvenience and, as demonstrated by other authors, the position has to be calibrated on the body habitus and after proper training on cadaveric models. This is far from the current robotic standard which is made of a linear trocar placement, fast docking and limited instruments fighting. Each multi-quadrant surgery has at least 3 different BSUs position to prevent and minimize the clashing. This could lead to the instrument blocking with a 20 s reset required and longer operative time. With the aim of avoiding the clashing, a person is required to monitor the robotic arms movements. Most of the time, after a proper countertraction applied by the fourth robotic arm, this was removed to reduce the fighting with the three other active arms.

The position of the BSUs around the table, despite their easy removal, does not allow the assistant surgeon to adopt a proper position. This is uncomfortable while performing high risk gestures as clipping or stapling.

The absence of dedicated energy devices reduces the procedural costs but does not allow a future adoption of robotic advanced bipolar energy. Furthermore, the actual robot allows for a maximum blended mode energy of 20 W. This low power requires longer time to perform safe and bloodless dissection.

A similar exists with respect to the insufflator which is not dedicated to reduce the cost of the procedure. The stability of the cavity is optimal but the absence of smoke evacuation raises questions about procedural safety and loss of time.

We were fully satisfied by the working posture of the master console architecture which has important implications for surgeons [18]. This new console is adjustable, reducing curved cervical posture and offering the possibility of a stand-up approach.

Conversely, we found a difficulty in the usage of the handles with an opposite movement of first and second finger to close the instruments’ jaws while activating the energy device.

The dexterity of the platform is not perfect in every situation and this was the reason for a missed intra-corporeal anastomosis due to the direction of the stumps compared to the instruments: when the suture is in sagittal position, it can be properly performed while different suturing directions put the surgeon in difficulty.

**Fig. 3:**
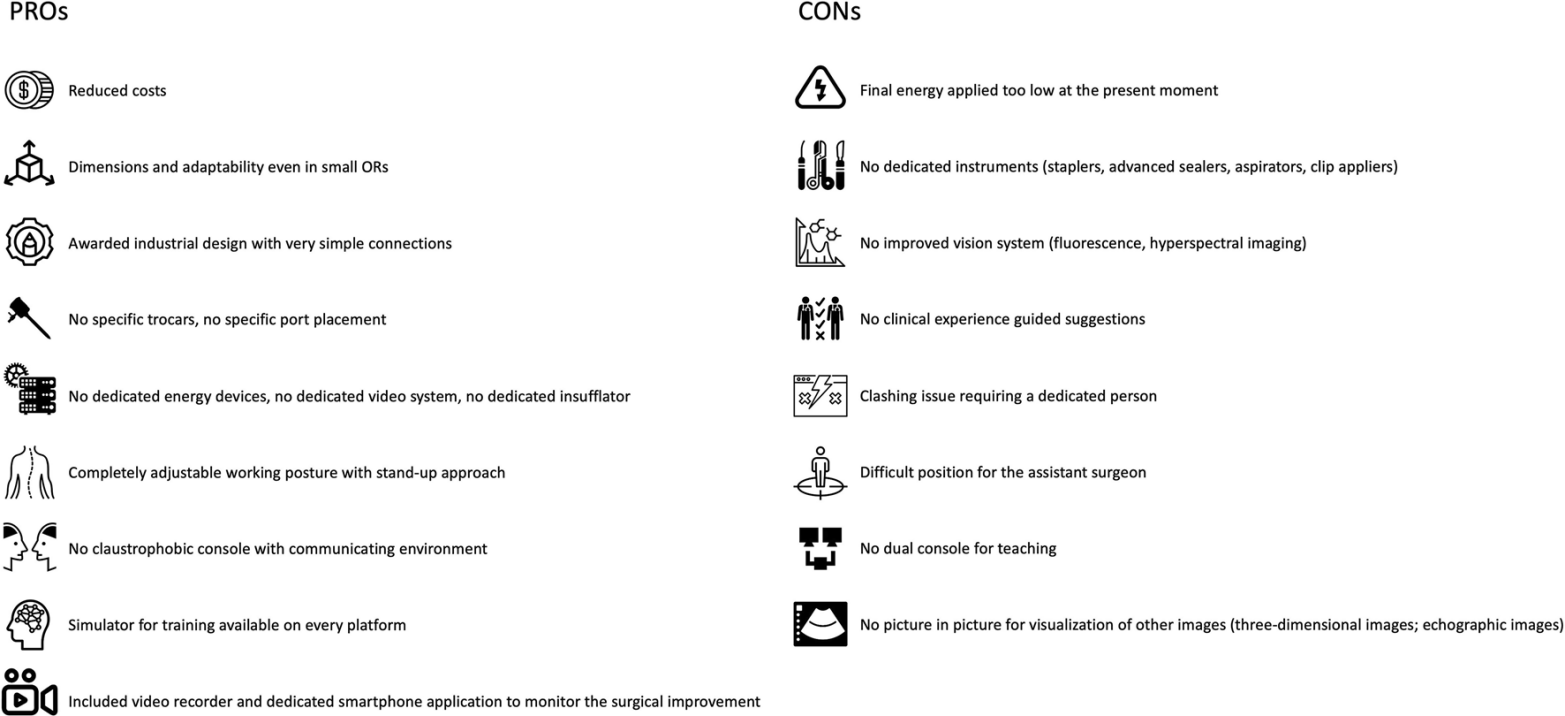
PROs and CONs of the robotic platform

The three-dimensional view guarantees high quality but the absence of advanced visualization systems is another weak point in the image guided surgery era. No near infrared imaging is available to adopt fluorescence guided surgery and no external video input is allowed for intraoperative navigation. Based on our experience, we summarise in Fig. 3 the previously discussed strengths and weaknesses of this new platform when performing robotic colorectal procedures.

Finally, our last concern regards the educational value of the robotic approach. The company provides a dedicated training program and a subsequent monitoring of the progression in surgery with a dedicated smartphone application. Unfortunately, to date, there are no dual console modalities or video highlighting functions to allow for surgical training.

## Conclusions

The present series confirms the feasibility of the full-robotic procedures while highlighting the strengths and the limits of the CMR Versius platform in colorectal surgery.

Further studies are needed to confirm the generalizability of the presented results. Improvements of the robotic platform are mandatory to standardize the colorectal procedures worldwide.

## Supplementary Information

Below is the link to the electronic supplementary material.

Supplementary file1 (MP4 84779 KB)

Supplementary file2 (MP4 92101 KB)

## Data Availability

Available data are reported in the present paper and further information can be requested in respect to the Italian privacy policy.
